# AMT-130 gene therapy: a promising disease-modifying approach for Huntington’s disease

**DOI:** 10.1097/MS9.0000000000004574

**Published:** 2025-12-10

**Authors:** Chisanga Mwape, Afnan Ahmad Qureshi, Muhammad Zaid Saeed, Aleeza Fatima, Aiman Jamil, Huzaifa Mahmood, Ailiya Batool, Abdul Moiz Khan

**Affiliations:** a Department of Internal Medicine, The Copperbelt University School of Medicine, Ndola, Zambia; b Department of Internal Medicine, Wah Medical College, Wah Cantt, Pakistan; c Department of Internal Medicine, Ayub Medical College, Abbottabad, Pakistan

**Keywords:** AMT-130, gene therapy, Huntington’s disease, microRNA, neurodegeneration

## Abstract

Huntington’s disease (HD) is a progressive, autosomal dominant neurodegenerative disorder caused by expanded Cytosine-Adenine-GuanineCAG repeats in the huntingtin (HTT) gene, leading to the production and accumulation of mutant huntingtin protein and subsequent neuronal dysfunction and loss. Current management remains largely symptomatic, with no established disease-modifying therapy. AMT-130 represents a novel and promising approach aimed at directly targeting the underlying molecular pathology of HD. AMT-130 is a one-time gene therapy that utilizes an adeno-associated virus serotype 5 (AAV5) vector to deliver an engineered microRNA (miHTT) into the caudate and putamen via stereotactic intracerebral infusion. This microRNA selectively reduces HTT mRNA levels, resulting in sustained lowering of mutant huntingtin protein. Preclinical studies in both large-animal and rodent models have demonstrated broad vector distribution, long-term expression, significant reduction in huntingtin levels, improved motor performance, decreased neuronal degeneration, and prolonged survival. Early Phase I/II clinical data indicate a favorable safety profile, reductions in neurofilament light chain levels, and stabilization of motor and functional decline, particularly in high-dose cohorts, suggesting a potential slowing of disease progression. While long-term efficacy and broader clinical validation are still required, AMT-130 shows strong potential to shift HD treatment from purely symptomatic care toward meaningful disease modification. Its success may also pave the way for microRNA-based therapies in other neurodegenerative disorders.

*To the Editor,*

This letter adheres to the TITAN 2025 guidelines for transparent reporting of artificial intelligence use in scientific communication[1].

Huntington’s disease (HD) is an autosomal dominant neurodegenerative disorder caused by expanded Cytosine-Adenine-Guanine (CAG) repeats in the huntingtin (HTT) gene, resulting in the production of mutant huntingtin protein. This toxic protein accumulates in neurons, particularly in the striatum and cortex, leading to progressive neuronal loss. Clinically, HD manifests as a triad of motor, cognitive, and psychiatric disturbances, including chorea, impaired executive function, and mood disorders. Symptoms typically begin in mid-adulthood and worsen over time, ultimately leading to severe disability and reduced life expectancy[2].

The pathophysiology of HD involves not only neuronal death but also synaptic dysfunction, impaired mitochondrial activity, and altered intracellular signaling, which together contribute to the complex clinical picture. Currently, there is no disease-modifying therapy; management remains symptomatic, addressing movement disorders, psychiatric symptoms, and cognitive decline[3].

Globally, HD is considered rare, but prevalence varies considerably. Estimates in Western countries range from 3 to 7 per 100 000, while East Asia and other regions report significantly lower rates, often <1 per 100 000, reflecting genetic differences, diagnostic capacity, and reporting practices[4]. Cognitive and psychiatric features often precede motor symptoms, underscoring the importance of early diagnosis and monitoring[5]. Despite decades of research, no disease-modifying therapy exists, and current management remains largely symptomatic, highlighting the urgent need for interventions targeting the underlying pathogenic mechanisms.

Against this background, AMT-130 emerges as one of the most promising disease-modifying interventions for HD. This therapy employs a one-time, stereotactic intracerebral infusion of an AAV5 vector encoding an engineered microRNA (miHTT) designed to reduce HTT mRNA levels and thereby lower mutant huntingtin protein. By delivering the vector directly into the caudate and putamen, the procedure circumvents the blood–brain barrier and enables lasting transduction of vulnerable neuronal populations. Extensive preclinical testing has demonstrated broad vector distribution, long-term miHTT expression, and sustained huntingtin suppression in large-animal models; in a transgenic HD minipig, intracranial AAV5-miHTT produced dose-dependent reductions in mutant HTT mRNA and protein throughout the brain, supporting translational feasibility[6].

Complementing large-animal data, rodent models have provided compelling proof of concept. In Q175 knock-in mice, a single AAV5-miHTT injection produced sustained reduction in HTT over 12 months, reduced aggregate formation, prevented neuronal dysfunction, improved motor coordination, and extended survival[7]. In another mouse model (Q175FDN), treatment with AAV5-miHTT led to dose-dependent reductions in mutant HTT, partial reversal of neurochemical abnormalities detected by magnetic resonance spectroscopy, and transcriptional rescue in the striatum, even when pathology was advanced[8].

Recent refinements in the design of miHTT have addressed pathogenic isoforms beyond full-length HTT: preclinical work targeting exon-1 HTT transcript (HTT1a) demonstrates that AAV5-miHTT can reduce both full-length and exon-1 HTT transcripts and proteins, which may enhance therapeutic benefit[9].

Although human clinical data remain preliminary, early Phase I/II results from AMT-130 treatment are promising. UniQure conducted a Phase I/II clinical study of AMT-130 in HD, and participants exhibited a favorable safety profile, reductions in neurofilament light chain (a biomarker of neurodegeneration), and stabilization of motor and functional performance. Remarkably, the high-dose cohort followed for three years showed an approximately 75% slowing of clinical decline compared with matched natural-history controls, providing one of the strongest signals yet for durable disease modification.

In conclusion, if these initial findings are replicated in larger, multicenter trials, AMT-130 could redefine HD treatment by shifting the paradigm from symptomatic care to sustained neuroprotection. Furthermore, its success could validate microRNA-based gene therapy as a powerful strategy for other neurodegenerative diseases. The field stands at a hopeful but cautious inflection point, one where cellular neuroscience, gene therapy, and patient advocacy converge to create genuine translational momentum.

## Data Availability

Not applicable.
